# Enhanced immune reaction resulting from co-vaccination of WT1 helper peptide assessed on PET-CT

**DOI:** 10.1097/MD.0000000000022417

**Published:** 2020-09-25

**Authors:** Jun Nakata, Kayako Isohashi, Soyoko Morimoto, Ryota Itou, Takashi Kamiya, Ai Matsuura, Hiroko Nakajima, Fumihiro Fujiki, Sumiyuki Nishida, Yoshiko Hasii, Kana Hasegawa, Shinichi Nakatsuka, Naoki Hosen, Akihiro Tsuboi, Yoshihiro Oka, Atsushi Kumanogoh, Masaru Shibano, Satoru Munakata, Yusuke Oji, Jun Hatazawa, Haruo Sugiyama

**Affiliations:** a Department of Clinical Laboratory and Biomedical Sciences; b Department of Nuclear Medicine and Tracer Kinetics, Osaka; c Department of Cancer Immunotherapy, Osaka University Graduate School of Medicine; d Department of Pathology, Sakai City General Hospital; e Department of Respiratory Medicine and clinical immunology, Osaka University Graduate School of Medicine; f Department of Hematology, Sakai City General Hospital; g Department of Cancer Immunology, Osaka University Graduate School of Medicine; h Department of Cancer Stem Cell Biology; i Department of Hematology and Oncology, Osaka University Graduate School of Medicine; j Department of Immunopathology, WP1 Immunology Frontier Research Center, Osaka University, Suita city, Osaka, Japan.

**Keywords:** cancer vaccine, FDG PET-CT, immunotherapy, WT1 vaccine therapy

## Abstract

It has become evident that positron emission tomography/computed tomography (PET-CT) using 2-deoxy-2-[F-18]fluoro-D-glucose (FDG) (FDG PET-CT) can detect anti-tumor immune response induced by various immunotherapies. To evaluate whether FDG PET-CT could detect anti-cancer immune response caused by cancer vaccine therapy, we performed a retrospective analysis of FDG PET-CT imaging of patients who were treated with Wilms Tumor 1 (WT1) vaccine therapy in Osaka University during July 2008 and June 2018. Increased FDG uptakes were detected in WT1-vaccinated skin and their draining lymph nodes during the repeated vaccination. While the FDG uptakes seemed to decrease with time after the cessation of WT1 peptide vaccinations, persistence of FDG uptakes for years in WT1-vaccinated skin were also observed in 2 cases who showed good clinical course. Moreover, the FDG uptakes of patients treated with the combination vaccine of WT1 specific cytotoxic T cell (CTL) and helper peptides were significantly stronger than of those treated with the WT1 CTL peptide alone. Since it is evident that the combination vaccine can induce a more robust anti-tumor immunity than can CTL peptide vaccine alone, the FDG uptakes in WT1-vaccinated skin might reflect the degree of immune response. These results suggest that PET-CT might be a good tool for prediction of anti-tumor immune response induced by WT1 vaccine therapy. Larger scale prospective studies therefore seem to be warranted.

## 1 Introduction

Positron emission tomography/computed tomography (PET-CT) using 2-deoxy-2-[F-18]fluoro-D-glucose (FDG) (FDG PET-CT) is an effective imaging technique for disease staging, detection of early disease recurrence and determining prognosis for many kinds of tumors.^[1,2]^ Furthermore, FDG PET-CT has recently been attracting attention as a tool for the detection of immune response induced by various kinds of immunotherapies. For example, administration of rituximab (anti-CD20 antibody) against B-cell lymphoma caused infiltration of lymphocytes into lymphoma lesions, and PET-CT imaging detected this as an immune flare in the lymphoma lesions.^[3]^ Vemurafenib, a BRAF inhibitor, which is a target therapy against BRAF-mutant melanoma, also induced T-cell immune response,^[4]^ and FDG uptake reflecting such immune response was seen in lymph nodes and spleen.^[5]^ Moreover, anti-CTLA4 therapy also induced immunological responses detectable by FDG PET-CT such as accumulation of activated lymphocytes in tumor sites.^[5,6]^ Such sensitive detection of immune response by immunotherapies was sometimes confusing as to whether the increased FDG uptake reflected the remaining tumor or strong anti-tumor immunity, because most of the immune response occurred in tumor sites. It is therefore important to integrate knowledge about how each immunotherapy affects the findings of FDG PET-CT imagings. Since no studies have been reported that evaluated FDG PET-CT imagings in patients who were treated with cancer vaccine therapy, we performed a retrospective analysis of the findings of the FDG PET-CT imagings for patients who had been enrolled in our previous clinical studies using Wilms Tumor 1 (WT1) vaccine therapy.^[7–11]^ In these clinical studies, WT1 vaccine therapy had been performed for cancer patients who had residual cancer lesion or high risk of relapse after initial therapy,^[9–12]^ and PET-CT imagings were taken to assess the residual tumor. Then, increased FDG uptake was detected in both vaccinated skin and its draining lymph nodes. While it is difficult to discriminate immune flare from relapse in lymph nodes, vaccinated skin should reflect only immune response activated by cancer vaccine therapy. We therefore evaluated the FDG uptake of the vaccinated skin in patients who were treated with WT1 vaccine therapy.

## 2 Materials and methods

### 2.1 Study design

The 2 cohort studies for this study were previously approved by the Institutional Review Board for Clinical Research, Osaka University Hospital. The former study was entitled with “Retrospective analysis of FDG uptake on PET-CT scan in patients treated with WT1 peptide vaccination”. In this prospective study, FDG PET-CT imaging history during July 2008 and June 2018 were examined for patients who had been enrolled in some kinds of clinical studies using WT1 vaccine therapy against malignancies in Osaka University.^[7–11]^ All clinical studies had been also approved by the Institutional Review Board for Clinical Research, Osaka University Hospital. From clinical records of patients who had performed FDG PET-CT in their clinical courses, patients characteristics, the period from vaccination to PET-CT scan, the maximum value of standardized uptake value (SUV) in the regions-of-interest (SUVmax) of the WT1-vaccinated skin areas assessed with FDG PET-CT and the types of WT1 vaccine therapy were taken out. Finally, a total of 37 PET-CT imagings of 24 patients were evaluated (Table 1). In all clinical studies, patients were continuously subcutaneously injected with WT1–126 peptide (RMFPNAPYL), modified WT1–235 peptide (CYTWNQMNL) or a cocktail of WT1–126, WT1–235, and WT1–332 (WAPVLDFAPPGASAYGSL) peptides combined with Montanide ISA51 adjuvant every 2 to 8 weeks. WT1–126 and WT1–235 peptides were cytotoxic T cell (CTL) peptides, and can induce peptide-targeted CTLs for patients with HLA-A 02;01 and 24;02, respectively. WT1–332 peptide was helper peptide, and can induce peptide-targeted helper CD4+ T-cell response. The cocktail of 3 peptides were named as Trio vaccine.

**Table 1:**
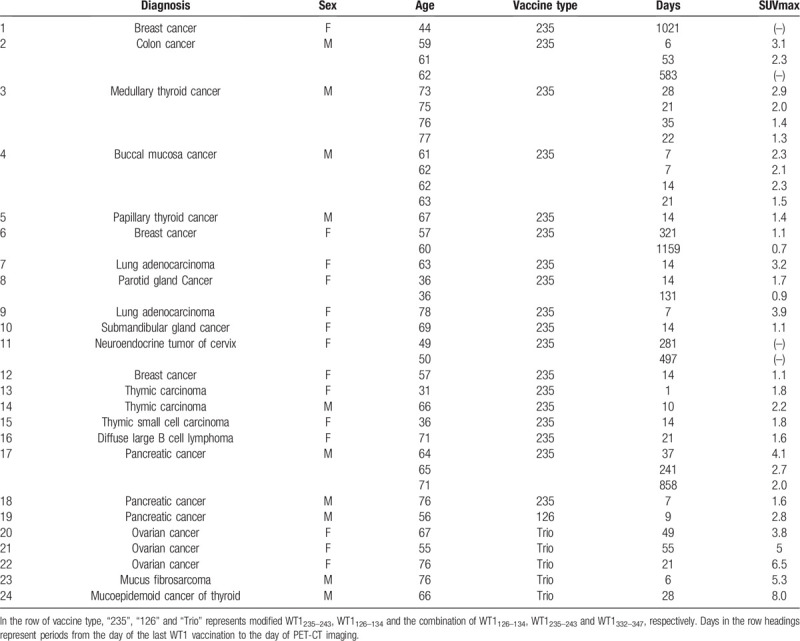
Patients characteristics and the maximum value of standardized uptake value (SUVmax) of their WT1-vaccinated skin areas assessed by positron emission tomography/computed tomography using 2-deoxy-2-[F-18]fluoro-D-glucose (FDG PET-CT).

The latter study was entitled with “Histological analysis of vaccinated skin of an autopsy patient”. In this study, histology of skin area vaccinated with WT1 Trio vaccine were examined. This patient had been enrolled in Phase I clinical study entitled with “multicenter study of WT1 Trio vaccine therapy against acute myeloid leukemia in 1st hematological complete remission after induction chemotherapy (UMIN 000017937)” in Sakai City General Hospital. This study was also approved by both the Institutional Review Board for Clinical Research, Osaka University Hospital and Sakai City General Hospital. In this study, WT1 Trio vaccine was scheduled to be subcutaneously injected twice every week before each consolidation chemotherapy. FDG PET-CT detected increased FDG uptakes on his vaccinated skin after 2nd WT1 peptide vaccination. Unfortunately, he died from a sepsis by Scedosporium prolificans during neutropenia after 4th consolidation chemotherapy, and autopsy was performed.

### 2.2 Hematoxylin and eosin (H&E) and immune-histochemical staining

Vaccinated skin from autopsy were fixed in 10% neutral buffered formaldehyde at room temperature for 24 to 48 hours and embedded in paraffin. For H&E staining, sections were deparaffinized and then stained with hematoxylin for 8 minutes. After washing with flowing water, they were stained with eosin for 2 minutes. For immunohistochemistry, stainings were performed on a BOND-III automated immunostainer (Leica Biosystems, Wetzlar, Germany). Sections were deparaffinized and then were pre-treated with pH 9.0 BOND Epitope Retrieval Solution 2 (Leica Biosystems) at 100°C for 20 minutes. Thereafter, they stained with ready to use anti-CD4 antibody (4B12: Leica Biosystems) or 1:100 anti-CD8 antibody (4B11: Leica Biosystems) or 1:50 anti-CD1 antibody (MTB1: Leica Biosystems) or 1:2 ready to use anti-CD20 antibody (L26: Nichirei Biosciences, Tokyo, Japan) or 1:150 anti-CD68 antibody (514H12: Leica Biosystems). Detection was performed using the BOND Polymer Refine Detection Kit (Leica Biosystems).

### 2.3 Statistical analysis

Two-sample *t*-test was used for the statistical analysis in all experiments. Differences were defined as statistically significant when *P* values were less than .05.

## 3 Results

### 3.1 Increased FDG uptakes reflecting immune response to WT1 vaccine therapy were detected in WT1-vaccinated skin and draining lymph nodes

We performed a retrospective analysis of FDG PET-CT imagings of patients treated with WT1 vaccine therapy in Osaka University during July 2008 and June 2018. A total of 37 PET-CT imagings of 24 patients were evaluated. Twenty nine of 37 PET-CT scans were performed during the repeated administration of WT1 vaccine therapy, and all of them showed measurable increases in FDG uptakes in the WT1-vaccinated skin (Table 1). The remaining 8 PET-CT imagings of 6 patients were obtained after discontinuation of the WT1 vaccine therapy, and half of them showed no FDG uptake. These results suggested that the FDG uptake of WT1-vaccinated skin seemed to decrease with time after the cessation of WT1 peptide vaccinations. Interestingly, FDG uptake in WT1-vaccinated skin continued for more than 858 days in patient 17 whose stage IV pancreatic cancer reached operable levels as a result of WT1 vaccine therapy and who was cured with a subsequent operation.^[11]^ The WT1-vaccinated skin of this patient could maintain its inflammatory status for years, and such strong and sustained immunity might be associated with the good clinical course.

**Figure 1:**
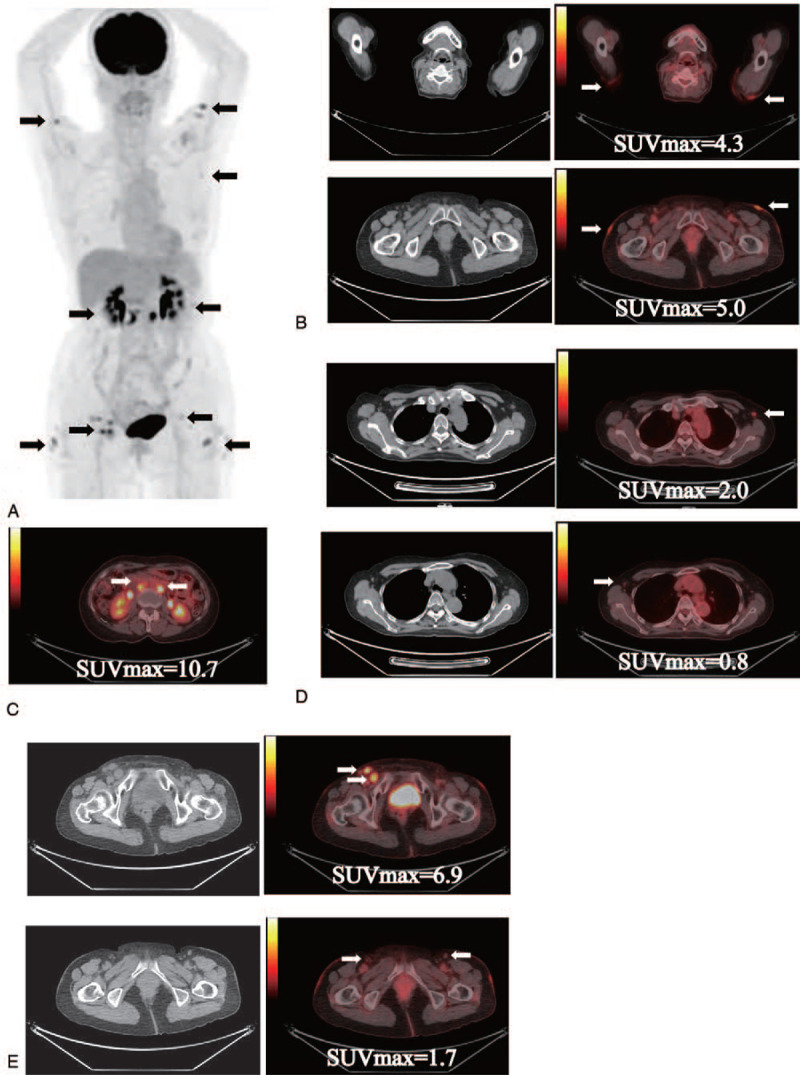
PET-CT imaging of an ovarian cancer patient treated with Trio vaccine. A 65-year-old female with an ovarian cancer underwent an extensive hysterectomy and subsequent chemotherapy. This was followed by continuous administration of the WT1 Trio vaccine therapy for the prevention of relapse. PET-CT imaging was performed as a routine check at the 17th months of the WT1 Trio vaccine therapy. (A) Axial image and coronal images of (B) skin of upper arm and femur, (C) para-aortic lymph nodes, (D) axillary lymph nodes and (E) inguinal lymph nodes. Arrows represent the regions of increased FDG uptakes.

There were also some patients who seemed to show increased FDG uptakes reflecting immune response in draining lymph nodes. For example, patient 21 was a 65-year-old female who was diagnosed with right ovarian cancer. Her tumor was 9.3 cm in diameter and had spread to the right external iliac artery lymph nodes and para-aortic lymph nodes (T1aN1M0). She underwent an extensive hysterectomy and was subsequently treated with a regimen of 6 courses of paclitaxel and carboplatin. Afterwards, she participated in a clinical study in which the WT1 Trio vaccine was administered for prevention of relapse. WT1 peptide vaccination was scheduled to continue for 2 years, and FDG PET-CT was performed 17 months after the start of WT1 vaccine therapy. The FDG PET-CT imagings showed enhanced FDG uptakes in para-aortic, bilateral inguinal, and bilateral axillary lymph nodes and the skin of upper arm and femur (Fig. 1A). The increased FDG uptakes of the WT1-vaccinated skin of upper arm and femur suggested there was an immune response resulting from theWT1 vaccine therapy, and SUVmax of the 2 areas were 4.3 and 5.0, respectively (Fig. 1B). On the other hand, SUVmax of para-aortic, bilateral inguinal and bilateral axillary lymph nodes were 10.7 (Fig. 1C), 0.8 and 2.0 (Fig. 1D), and 6.9 and 1.7 (Fig. 1E), respectively. As described in Discussion and conclusions section, these FDG uptakes seemed to reflect a mixture of relapsed and reactive lymph nodes.

### 3.2 Increased FDG uptake of vaccinated skin was much stronger in patients treated with combination vaccine of WT1-CTL and helper peptides than those with the WT1-CTL peptide vaccine alone

**Figure 2:**
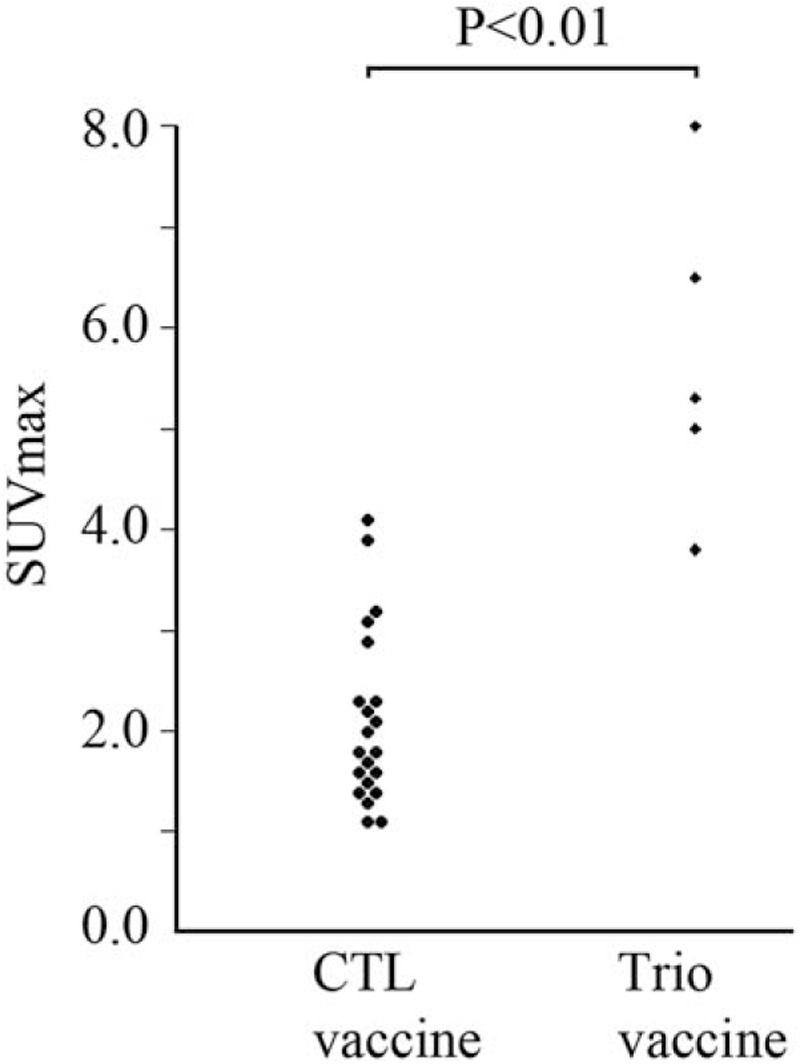
Evaluation of associations between SUVmax of WT1-vaccinated skin and clinical parameters. Beeswarm plot of SUVmax of WT1-vaccinated skin in relation to the type of WT1 cancer vaccine.

Next we evaluated whether SUVmax of WT1-vaccinated skin reflect the magnitude of anti-tumor immune response. This study contained both very slow and very fast progressive tumors, and also includes cases in which WT1 vaccine therapy succeeded in suppressing tumor progression but FDG PET-CT was performed because tumor progressed thereafter. Also, the timing of FDG PET-CT varied with individual cases. Therefore, it was difficult to define the clinical status at the timing of FDG PET-CT. Then, the association of the SUVmax with types of peptides used for WT1 vaccine therapy was analyzed (Fig. 2). SUVmax of vaccinated skin was significantly higher in patients treated with combination vaccine than those with WT1-CTL vaccine alone. Especially, the weakest FDG uptake in patients treated with combination vaccine was nearly same level as the strongest FDG uptake in patients with CTL peptide vaccine. Since the addition of WT1 helper peptide on WT1-CTL peptide vaccination were reported to induce stronger WT1-specific CTL response and better clinical outcome,^[13]^ these results suggested the possibility that increased FDG uptake of WT1-vaccinated skin assessed by PET-CT reflect the magnitude of anti-cancer immunity induced by WT1 vaccine therapy.

### 3.3 CD4+, CD8+ T cells and CD68+ macrophages mainly infiltrated at the WT1-vaccinated skin showing increased FDG uptake by FDG PET-CT

**Figure 3:**
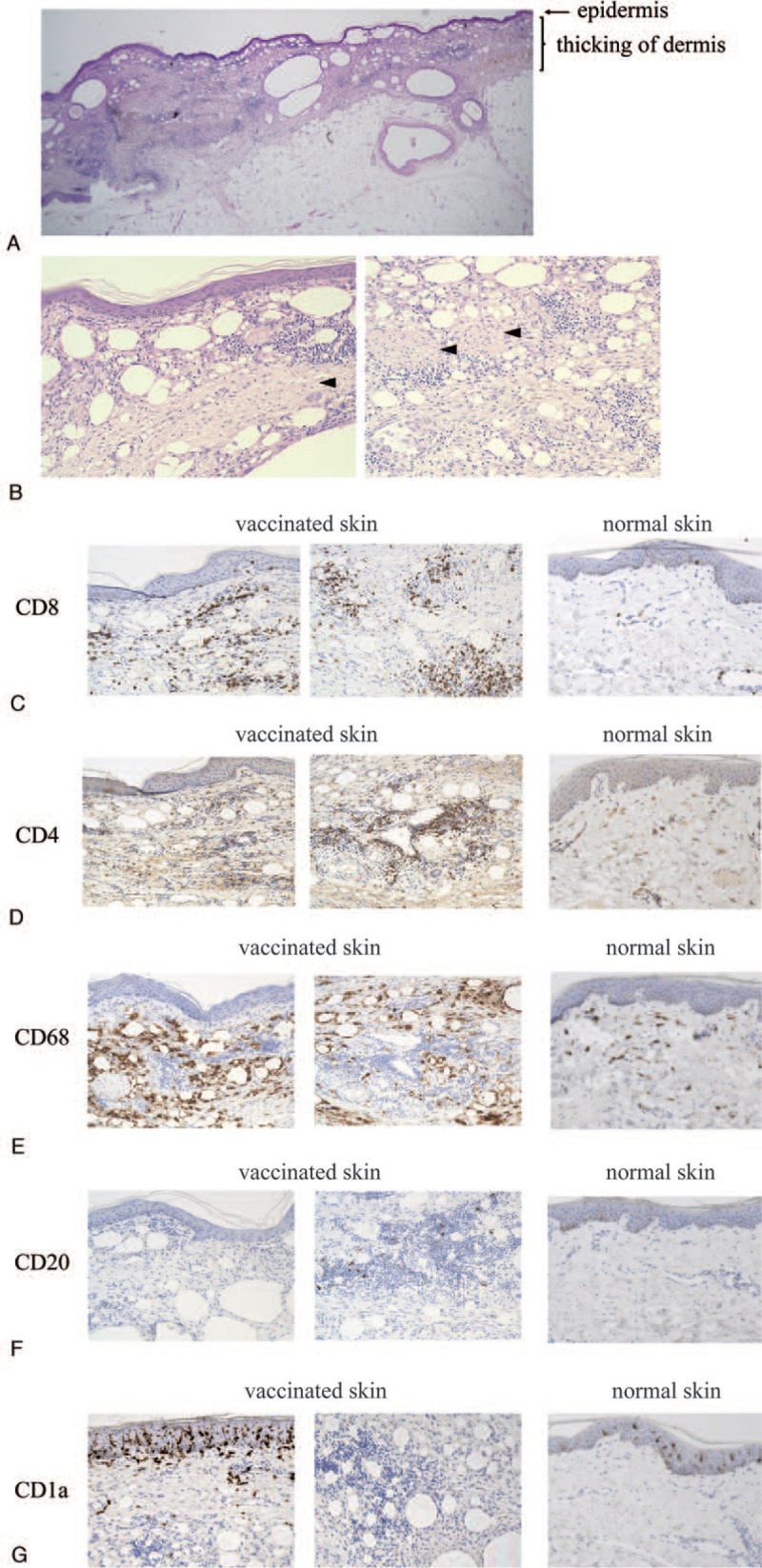
Histological examination of WT1-vaccinated skin from an autopsied patient treated with Trio vaccine. (A) Low-power field of H&E staining of WT1-vaccinated skin. (B) High-power field of H&E staining of WT1-vaccinated. The arrow in the left figure represents fibrotic change, and the arrows in the right figure represent granuloma formation. (C-G) Images of immunostaining of WT1-vaccinated skin and normal skin for CD8 antibody (C), CD4 antibody (D), CD68 antibody (E), CD20 antibody (F) and CD1a antibody (G).

WT1-vaccinated skin where FDG PET-CT detected the increased FDG uptakes was also histologically analyzed from a patient with autopsy. H&E staining of WT1-vaccinated skin, which were injected intradermally 2 months before his death, showed the thickening of dermal layer (Fig. 3A). Fibrosis and granuloma formation were seen in some areas. And, the drops of Montanide ISA 51 accompanied with the infiltration of many immune cells were also seen in the thickening of dermal layer (arrows in Fig. 3B). Next, immune-histochemical staining by CD4, CD8, CD68, CD20, and CD1a antibodies were performed. Both CD4+ and CD8+ T cells widely infiltrated in all areas and also formed cell-clusters in some areas (Fig. 3C and D). CD68+ macrophages infiltrated and also surrounded some of the Montanide ISA 51 drops (Fig. 3E). On the contrary, CD20+ B cells were rarely seen in very limited area (Fig. 3F). And, CD1a+ Langerhans cells were mostly seen in epidermis, not in the thickening of dermis (Fig. 3G). These results suggested that CD4+, CD8+ T cells, and CD68+ macrophage cells mainly infiltrated into WT1-vaccinated skin, and that FDG PET-CT imagings might detect immune response by them.

## 4 Discussion and conclusions

The present study is the first report of FDG PET-CT imagings in patients treated with cancer vaccinations. Increased FDG uptakes were detected both in WT1-vaccinated skin and their draining lymph nodes. Especially, all patients showed increased FDG uptakes in the WT1-vaccinated skin during the vaccination. Histologically, CD4+ or CD8+ T cells, and CD68+ macrophages were infiltrated in these WT1-vaccinated skin. We knew that cancer vaccinations with Montanide ISA 51 lead grade 1–3 injection site reaction,^[9]^ and our results were clear proofs that these injection site reaction reflected the local immune response.

As for FDG uptakes in lymph nodes, it was often difficult to distinguish immune response from tumor relapse. For example, in a patient shown in Figure 1, the para-aortic lymph nodes were not regional lymph nodes of the sites where WT1 peptide vaccination was administered but those of the primary tumor sites. In addition, cancer antigen 125 levels were also elevated. Furthermore, the SUVmax was 10.7, which was also much higher than that of the reactive lymph nodes (Fig. 1C). These increases in FDG uptakes of the para-aortic lymph nodes should therefore have been due to relapse. As for the bilateral axillary lymph nodes that were regional lymph nodes vaccinated with the WT1 peptide, CT images showed that both left and right lymph nodes were less than 10 mm in diameter along the short axis and were crescent shaped with smooth margins (Fig. 1D). Both features are commonly seen in reactive lymph nodes. In addition, the SUVmax of the left and right lymph nodes was low (Fig. 1D; 0.8 and 2.0, respectively), while the relapse was localized below the diaphragm and never extended as far as above the diaphragm for more than a year after the FDG PET-CT imaging was obtained. When these findings are put together, it appears that these axillary lymph node swellings were due to immunological response generated by WT1 vaccine therapy rather than to relapse. In view of these observations, the increases in the FDG uptakes of the bilateral inguinal lymph nodes might be due to a mixture of relapse and reactive lymph nodes. Some of the lymph nodes showed high levels of FDG uptake (SUVmax 6.9), while others showed low levels (SUVmax 1.7) with crescent shapes and smooth margins (Fig. 1E). These results suggest that both relapse and anti-tumor immune response induced by WT1 vaccine therapy should be considered when increased FDG uptakes were detected in lymph nodes, and also suggested that their shapes and SUVmax might be useful for discrimination.

The addition of WT1 helper peptide on WT1-CTL peptide vaccination induced higher anti-tumor immunity.^[13,14]^ And SUVmax of WT1-vaccinated skin, which might reflect the magnitude of immune reaction of this lesion, were significantly higher in patients treated with WT1 Trio vaccine than those with WT1-CTL vaccine. In addition, in patient 17 whose stage IV pancreatic cancer shrieked to the operable levels by the WT1 peptide vaccination and was cured by the subsequent operation,^[11]^ SUVmax of WT-vaccinated skin was more than 2.0 continued for 858 days. The long persistence of immune reaction by the WT1 vaccination might be associated with his good clinical course. These findings suggested that the values of FDG uptake of WT1-vaccinated skin could reflect the magnitude of systemic immune response induced by WT1 vaccine therapy. FDG PET-CT could thus be useful not only for assessment of the quantity and extent of remaining tumors but also to estimate the magnitude of the immune response generated by cancer vaccine. To validate our findings, however, larger prospective studies are warranted.

## Author contributions

**Conceptualization:** Jun Nakata.

**Data curation:** Jun Nakata, Kayako Isohashi, Ryota Itou, Takashi Kamiya, Ai Matsuura, Hiroko Nakajima, Fumihiro Fujiki, Sumiyuki Nishida, Satoru Munakata.

**Formal analysis:** Jun Nakata, Soyoko Morimoto, Ryota Itou, Takashi Kamiya, Ai Matsuura, Hiroko Nakajima, Fumihiro Fujiki, Satoru Munakata.

**Funding acquisition:** Yoshihiro Oka, Yusuke Oji, Haruo Sugiyama.

**Investigation:** Jun Nakata, Kayako Isohashi.

**Methodology:** Jun Nakata.

**Project administration:** Jun Nakata.

**Supervision:** Akihiro Tsuboi, Yoshihiro Oka, Atsushi Kumanogoh, Masaru Shibano, Yusuke Oji, Jun Hatazawa, Haruo Sugiyama.

**Validation:** Ai Matsuura, Yoshiko Hasii, Kana Hasegawa, Shinichi Nakatsuka, Naoki Hosen, Akihiro Tsuboi, Masaru Shibano.

**Writing – original draft:** Jun Nakata, Yoshihiro Oka, Atsushi Kumanogoh, Jun Hatazawa, Haruo Sugiyama.

**Writing – review & editing:** Jun Nakata, Shinichi Nakatsuka, Yoshihiro Oka, Haruo Sugiyama.
